# Evaluating the Benefit of Home Support Provider Services for Positive Airway Pressure Therapy in Patients With Obstructive Sleep Apnea: Protocol for an Ambispective International Real-World Study

**DOI:** 10.2196/65840

**Published:** 2025-01-31

**Authors:** Sarah Alami, Manuella Schaller, Sylvie Blais, Henry Taupin, Marta Hernández González, Frédéric Gagnadoux, Paula Pinto, Irene Cano-Pumarega, Lieven Bedert, Ben Braithwaite, Hervé Servy, Stéphane Ouary, Céline Fabre, Fabienne Bazin, Joëlle Texereau

**Affiliations:** 1 Air Liquide Santé International Bagneux France; 2 VitalAire Spain Madrid Spain; 3 Department of Respiratory and Sleep Medicine Angers University Hospital Angers France; 4 Thorax Department Unidade Local de Saúde Santa Maria Lisbon Portugal; 5 Faculty of Medicine of Lisbon Instituto de Saúde Ambiental Lisbon Portugal; 6 Sleep Unit and Respiratory Department Hospital Ramón y Cajal Instituto Ramón y Cajal de Investigación Sanitaria Madrid Spain; 7 Department of Respiratory Medicine Ziekenhuisnetwerk Antwerpen Middelheim Hospital Anvers Belgium; 8 Sanoïa Aubagne France; 9 Horiana Bordeaux France; 10 Air Liquide Healthcare Bagneux France; 11 Cochin University Hospital Assistance Publique - Hôpitaux de Paris Paris France

**Keywords:** obstructive sleep apnea, positive airway pressure, real-world evidence, home support provider, adherence, electronic patient-reported outcome, comparative real-world study

## Abstract

**Background:**

Adherence and persistence to positive airway pressure (PAP) therapy are key factors for positive health outcomes. Home support providers participate in the home implementation and follow-up of PAP therapy for patients with obstructive sleep apnea (OSA). In Europe, home support provider service levels are country (or area) specific, resulting in differences in content and frequency of patient interactions. However, no robust evaluation of the impact of these differences on clinical and patient outcomes has been performed.

**Objective:**

The AWAIR study aims to evaluate and compare the impact of different home support provider service levels on PAP adherence and persistence in 4 European countries.

**Methods:**

This real-world, ambispective, cohort study—conducted in France, Belgium, Spain, and Portugal—will recruit adults with OSA who started PAP therapy between 2019 and 2023 and were followed by an Air Liquide Healthcare home support provider. Given the large number of eligible participants (around 150,000), the study will use a decentralized and digital approach. A patient video will present the study objectives and the participation process. A secure electronic solution will be used to manage patient information and consent, as well as to administer a web-based questionnaire. Retrospective data, collected during routine patient follow-up by home support providers, include the level of service and device data, notably PAP use. Prospective data collected using an electronic patient-reported outcome tool include health status, OSA-related factors, patient-reported outcomes including quality of life and symptoms, OSA and PAP literacy, patient-reported experience, and satisfaction with PAP therapy and service. Hierarchical models, adjusted for preidentified confounding factors, will be used to assess the net effect of home support provider services on PAP adherence and persistence while minimizing real-world study biases and considering the influence of country-level contextual factors. We hypothesize that higher levels of home support provider services will be positively associated with adherence and persistence to PAP therapy.

**Results:**

As of December 2024, the study has received approval in France, Portugal, and 2 regions of Spain. The study began enrollment in France in October 2024. Results are expected in the second quarter of 2025.

**Conclusions:**

The AWAIR study has a unique design, leveraging an unprecedented number of eligible participants, decentralized technologies, and a real-world comparative methodology across multiple countries. This approach will highlight intercountry differences in terms of patient characteristics, PAP adherence, and persistence, as well as patient-reported outcomes, patient-reported experiences, and satisfaction with the home service provider. By assessing the added value of home support provider services, the results will support best practices for patient management and for decision-making by payers and authorities.

**International Registered Report Identifier (IRRID):**

PRR1-10.2196/65840

## Introduction

Obstructive sleep apnea (OSA) is the most prevalent form of sleep-disordered breathing, affecting nearly 1 billion adults aged 30-69 years worldwide [1]. OSA is characterized by repetitive collapse of the upper airway during sleep, resulting in complete (apnea) or partial (hypopnea) airway obstruction [2]. OSA can cause daytime symptoms that impair quality of life and has been linked with a variety of cardiometabolic diseases, neurocognitive impairment, depression, road traffic accidents, and all-cause mortality [3-12]. Identification and management of OSA are therefore important to reduce those risks.

Positive airway pressure (PAP) therapy remains the gold standard treatment for severe OSA because high-quality evidence and long-term assessment are lacking for noncontinuous PAP therapies [13,14]. PAP therapy has been shown to reduce road traffic accidents, blood pressure, cardiovascular risk, and health care use [15-19]. However, the effectiveness and cost-effectiveness of PAP therapy are linked to sufficient daily device use, with regular and continued long-term usage [16,19-24], which appears challenging in all geographies [25,26].

In most European countries, home support provider companies supply the PAP device and support its use by the patient. However, the type and level of services provided differ markedly between countries. Services can range from mainly logistical support (ie, device delivery in Belgium, Germany, and the Netherlands) to a personalized support plan for patients integrating motivational interviews to enhance adherence (as in some regions of Spain and Portugal) [27-29]. Also, the number of regulated home visits varies from none to several per year (like in France and Portugal). The use of PAP telemonitoring also varies widely, being routine for almost all patients in France throughout the PAP treatment period [30] but not used (or only used in the first months of therapy or in specific situations) in other countries. Criteria for the reimbursement of PAP therapy also differ markedly between European countries and are likely to influence adherence and persistence. For example, while the fees paid to the home support providers in France increase as the number of hours of PAP use increases [30,31], in Belgium and Germany, nonadherence can result in the cessation of patient reimbursement.

The large number of patients treated with PAP therapy and the heterogeneity of home support provider service levels across Europe provide a unique opportunity to evaluate the optimal management approach. This requires an assessment of the added value of the service and of its impact on PAP adherence and persistence; quality of life; patient-reported outcomes (PROs), including quality of life and symptoms, patient-reported experience (PREs), and satisfaction.

Air Liquide Healthcare (ALH) is composed of several home support provider companies operating in more than 40 countries worldwide and 10 European countries. Support to patients with sleep apnea requiring PAP treatment is provided in all these countries. ALH designed the AWAIR (Assessment of the Benefit of the Home Support Provider Service Level Associated With Positive Airway Pressure Treatment in Patients With OSA on Treatment Adherence and Persistence: an International Real-World) study to assess and compare PAP adherence and persistence across 4 different European countries and according to the level of service. The study will consider the main confounding factors for adherence and persistence to PAP therapy and will provide unique information about differences in patient profiles across countries, PROs, OSA and PAP literacy, and experience and satisfaction with PAP therapy and service (PREs).

## Methods

### Study Design

This international, ambispective, real-world, cohort study is being conducted in 4 European countries (France, Belgium, Spain, and Portugal). It includes a retrospective analysis with the reuse of home support provider data routinely collected for PAP-related services and a prospective evaluation with the collection of patient data using a web-based patient questionnaire.

### Study Objectives

The main objective of the study is to compare PAP adherence and the PAP therapy persistence rate between countries and between different levels of home support provider service.

The study’s other objectives are to (1) identify service components that influence PAP adherence and persistence; (2) identify factors associated with PAP adherence and persistence; (3) describe in each country sociodemographic and OSA-related clinical characteristics, home support provider PAP-related service, OSA and PAP literacy of participants (perceived and actionable competence), patient experience and satisfaction with PAP therapy and home support provider support, patient-reported OSA symptoms, and quality of life (determined using the EQ-5D-5L questionnaire [32]); and (4) describe patterns of PAP use.

### Participants

#### Process for Selecting Patients

Eligible participants are adults (aged 18 years and older) who started PAP therapy between January 1, 2019, and December 31, 2023; for whom PAP services were delivered by an ALH affiliate in France, Belgium, Spain, or Portugal; and had a valid email address or smartphone number.

Potential participants were identified by applying these inclusion criteria to the data sourced from each home support provider database. In France and Portugal, all patients who met the above inclusion criteria were informed about the study. In Spain and Belgium, which have regional- or hospital-level regulatory processes, potential participants are only included from representative investigational sites. After providing the information about the study, patients who either consented to participate (Portugal, Spain, and Belgium) or did not “opt-out” (France) are enrolled in the study. These processes are described in more detail in the Ethical Considerations section.

The digital and decentralized nature of the study using e-consent and an electronic patient-reported outcome (ePRO) tool means that patients are not directly recruited by investigators. Instead, a secure electronic solution, Dr Data Consent, is used to provide direct and individual information about the study, obtain consent, and provide access to the web-based questionnaire. This solution is compliant with European and French legislation and regulations on the protection of personal data, as well as ethical guidance about patient’s rights management. Its technology is powered by blockchain thus guaranteeing full traceability on e-consents.

#### Process for Contacting and Recruiting Patients

The list of eligible patients that was extracted from each home support provider database contains patient contact details (ie, email address or smartphone number). This list is uploaded into Dr Data Consent and an algorithm checks the validity of the contact details. Eligible patients with valid contact information receive an email or SMS invitation to connect to this platform for the consent process. The invitation does not include sensitive data.

An explanatory video is provided, within the Dr Data Consent account of each patient, to ensure that potential participants fully understand the study’s aims, procedures, and their rights.

Those who provide consent are granted access to the ePRO tool and invited to complete the questionnaire, which should take them an estimated 10 minutes.

### Ethical Considerations

The study protocol was developed in collaboration with a scientific committee composed of experts in OSA and methodologists from the 4 countries involved. The regulatory process and patient consent for a study with the reuse of data and a questionnaire were different for the 4 countries.

In France, the study followed the methodology MR-004 of the French Data Protection Authority (Commission Nationale Informatique et Liberté) concerning research that reuses data that have already been collected [33] and was approved by the local ethics committee of Angers University Hospital in November 2023 (2023-159). In Portugal, the study was considered observational and required approval by a central ethics committee; the approval was obtained from the ethics committee of Lisboa in May 2024 (24/24). In Spain, the study was considered observational and required approval by the ethics committee at each participating hospital; approval was obtained from the CEIm Hospital Universitario Ramón y Cajal of Madrid in July 2024 (095/24) and from the CEIm Hospital Universitario Doctor Peset of Valencia in December 2024 (127/24) and is pending for other regions. In Belgium, the study was considered interventional and required approval by a central ethics committee (Commissie voor Medische Ethiek Ziekenhuisnetwerk Antwerpen Institutional Review Board, Ziekenhuis aan de Stroom Middelheim) and by the local ethics committee at each participating hospital. Ethics approvals from the belgian central ethics committee are still pending.

In France, potential participants have the option to decline the reuse of their personal data through an “opt-out” process. Conversely, in Belgium, Portugal, and Spain, an “opt-in” process has been established, requiring explicit consent from participants to join the study.

No compensation is provided to participants.

### Outcome Measures

Study outcomes, time points, and data sources are summarized in Table 1. An ePRO tool will be used to administer the patient questionnaire. The ePRO tool was designed to assess various aspects of a patient’s status, condition, and experience at 2 time points: the time of ePRO completion and the beginning of PAP therapy. This included sociodemographic characteristics (age, relationship status, employment, education level, and social support), health status (BMI, smoking status, alcohol intake, comorbidities, sleep duration, and use medications to aid sleep), OSA-related factors (when PAP therapy was started, apnea-hypopnea index before starting PAP, and OSA symptoms), PAP therapy experience and satisfaction (tolerability of PAP, attitude to PAP, device noise, and level of motivation to use PAP), patient experience and satisfaction with home support providers, quality of life (including current health status), and OSA-PAP literacy. The full ePRO questionnaire is provided in Multimedia Appendix 1.

Home support provider service will be assessed using 4 components: frequency of interactions between the home support provider and a patient; content of support (logistical, educational, supportive, or behavioral); personalization of support according to patient profile or needs; and telemonitoring (telemetry, data visualization, and alerts triggering home support provider interventions).

**Table 1:** Study outcomes.

Category and outcome		Time points	Source
**Sociodemographic and OSA^a^-related clinical characteristics**			
	Demographics (age and sex) BMI	At the start of PAP^b^ therapy	ALH^c^ ERP^d^
	Occupational status and highest education level Sleep characteristics: bed sharing, sleep duration, and sleep medication Smoking status and alcohol consumption BMI	At the time of ePRO^e^ completion	ePRO tool
	Comorbidities: diabetes, stroke, myocardial infarction, coronary stenting or heart surgery, heart failure, cardiac arrhythmia, hypertension, GORD^f^, COPD^g^, asthma, chronic rhinitis, cancer, anxiety or depression, and insomnia	Before starting PAP therapy	ePRO tool
	Apnea-hypopnea index at the time of diagnosis	Before starting PAP therapy	ALH ERP and ePRO tool
**PROs^h^**			
	Symptoms: snoring, excessive daytime sleepiness, daytime fatigue, lack of energy, waking up feeling tired, poor sleep, falling asleep at the wheel, nocturia, abrupt awakening with choking and gasping, morning headache, irregular breathing during sleep, decreased libido, depression, anxiety, irritability, and memory or concentration difficulties	Before and after starting PAP therapy	ePRO tool
	Quality of life using the EQ-5D-5L [32]	At the time of ePRO completion	ePRO tool
**OSA and PAP literacy**			
	Perceived and actual competence related to disease literacy and PAP use assessed using 13 variables with binary responses	At the time of ePRO completion	ePRO tool
**Experience and satisfaction with PAP therapy**			
	Measured using a visual analogue scale for Difficulty tolerating PAP (discomfort related to pressure, and mask) Likelihood of continued use Perceived health benefits Global satisfaction Noise acceptance/impact Motivation to use	At the time of ePRO completion	ePRO tool
**Experience and satisfaction with home support provider**			
	Measured using a 5-point Likert scale for Home support provider availability for logistical support Home support provider availability for educational support Satisfaction with home support provider Perceived value of home support provider	At the time of ePRO completion	ePRO tool
**Characteristics of PAP therapy**			
	Type of PAP device and mask	At the start of PAP therapy	ALH ERP
	Elevated apnea-hypopnea index Elevated unintentional leaks	1, 4, 6, and 12 months after starting PAP therapy and annually thereafter	Device data
**Home support provider PAP-related service**			
	Number and type of interactions (home visit, phone call, and digital interaction) Number of mask changes Implementation of telemonitoring	1, 4, 6, and 12 months after starting PAP therapy and annually thereafter	ALH ERP
**PAP adherence**			
	Mean number of hours per day of PAP use Mean PAP use categorized as: 0-2 hours/day, 2-4 hours/day, 4-6 hours/day, and ≥6 hours/day Proportion of adherent patients defined as (1) mean PAP use ≥4 hours/day, (2) mean PAP use ≥4 hours/day on ≥70% of days, and (3) mean PAP use ≥4 hours/day on ≥80% of days	1, 4, 6, and 12 months after starting PAP therapy and annually thereafter	Device data
**PAP persistence**			
	Percentage of days with or without PAP use defined as the ratio between the number of days with PAP usage (starting from 1 minute of use) over the number of days in the follow-up period Patients without PAP discontinuation (still in therapy), with temporary discontinuation, or with permanent discontinuation Patients still in therapy and with mean PAP use ≥4 h/day	4, 6, and 12 months after starting PAP therapy and annually thereafter	Device data and ALH ERP

^a^OSA: obstructive sleep apnea.

^b^PAP: positive airway pressure.

^c^ALH: Air Liquide Healthcare.

^d^ERP: Enterprise Resource Planning.

^e^ePRO: electronic patient-reported outcome.

^f^GORD: gastro-esophageal reflux disease.

^g^COPD: chronic obstructive pulmonary disease.

^h^PRO: patient-reported outcome.

### Data Sources, Flow, and Management

Potential participants will be identified by applying the inclusion criteria to the data sourced from the ALH Enterprise Resource Planning (ERP) systems. Initially, potential participants will receive an email or SMS invitation to connect to a secure platform for the consent process. Those who provide consent will be granted access to the ePRO tool and will be invited to complete the questionnaire, which takes about 10 minutes.

The study database will be created from 3 data sources: the ALH ERP systems that contain data routinely collected for PAP-related services; the database containing information about PAP device use (collected remotely through the Internet of Things or by download from device memory cards during home provider support visits); and the ePRO tool. Database structure and content will be standardized across countries.

Because the ALH ERP and PAP device use databases are primarily designed for administrative, logistical, and patient follow-up purposes, rather than research, all data will undergo thorough review for completeness, accuracy, and reliability before statistical analysis. These reviews will be conducted in accordance with a detailed data management plan, involving multiple experts with complementary areas of expertise. All decisions made during the review process will be fully documented.

**Figure 1:**
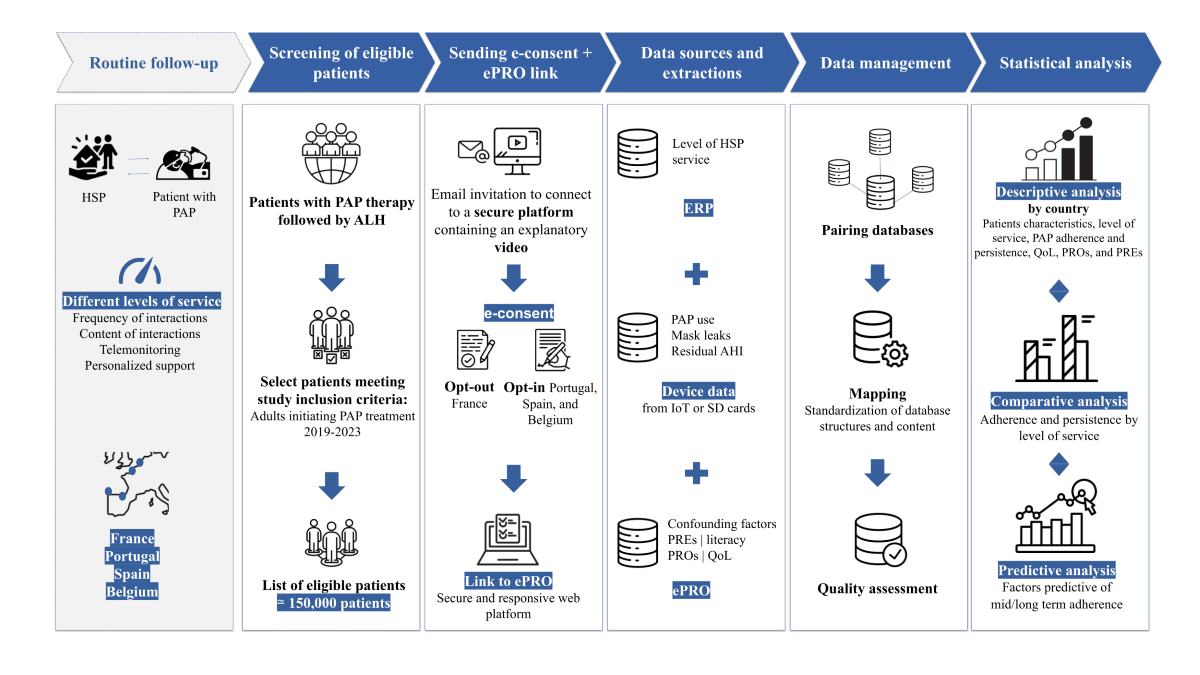
Diagram of data sources and data flow. AHI: apnea-hypopnea index; ALH: Air Liquide Healthcare; ePRO: electronic patient-reported outcome; ERP: Enterprise Resource Planning; HSP: home support provider; IoT: Internet of Things; PRE: patient-reported experience; QoL: quality of life.

All identifiable personal information will be protected and stored in the study database in pseudonymized form so that it cannot be viewed by the study sponsor or any external vendors that are processing the study data. A summary of data sources and data flow is provided in Figure 1.

### Statistical Methods

#### Sample Size

This study aims to include all adults starting PAP therapy managed by an ALH home support provider in the participating countries who met the selection criteria during the study time period. This approach minimizes selection bias and sampling errors, ensuring precise and reliable results. Inclusions will be possible at the national level (France) or only at some investigational sites in countries requiring regional or hospital-level regulatory processes. All potential participants will be informed whenever ethics committee approval is obtained from the respective regions or hospitals. It is expected that around 30% of the 150,000 eligible participants will not be enrolled due to the absence of (or incorrect) email, or refusal to participate. Therefore, the number of included patients is expected to be around 100,000. With a sample of this size, the probability of showing a statistically significant difference in adherence between different levels of service if one exists is >99%, even if there were important imbalances between groups.

#### Statistical Analysis

Statistical analyses will be performed according to a predefined statistical analysis plan (Table 2).

**Table 2:** Statistical analysis plan details.

Objectives	Main outcomes	Key explanatory variables	Statistical methods
Compare PAP^a^ adherence and the PAP therapy persistence rate between different levels of home support provider service	PAP adherence: (1) mean number of hours/day of PAP use and (2) adherent patients defined as those with mean PAP use ≥4 hours/day PAP persistence: (1) percentage of days with PAP use, (2) patients still in therapy, and (3) patients still in therapy and adherent	Fixed effect: Level of service (standard of care, personalized care plan, or high service level) Random effect: Country	Multilevel model adjusted for selected confounding factors
Identify service components that influence PAP adherence and persistence	PAP adherence: (1) mean number of hours/day of PAP use and (2) adherent patients defined as those with mean PAP use ≥4 hours/day PAP persistence: (1) percentage of days with PAP use, (2) patients still in therapy, and (3) patients still in therapy and adherent	Fixed effect: Frequency of interactions, content of support, personalization of support, and telemonitoring Random effect: Country	Multilevel model adjusted for selected confounding factors
Identify factors associated with PAP adherence and persistence	PAP adherence: (1) mean number of hours/day of PAP use and (2) adherent patients defined as those with mean PAP use ≥4 hours/day PAP persistence: (1) percentage of days with PAP use, (2) patients still in therapy, and (3) patients still in therapy and adherent	Fixed effect: Frequency of interactions, content of support, personalization of support, telemonitoring, sociodemographic characteristics, OSA^b^-related clinical characteristics, and characteristics of PAP therapy Random effect: Country	Multilevel model
Descriptive Analysis	Sociodemographic characteristics OSA-related clinical characteristics OSA and PAP literacy Experience with PAP therapy Experience with home support provider support Quality of life Home support provider PAP-related service	N/A^c^	Usual descriptive statistics: Analysis by country
Describe patterns of PAP use	Mean PAP use categories over time	N/A	Data visualization such as Sankey diagrams

^a^PAP: positive airway pressure.

^b^OSA: obstructive sleep apnea.

^c^N/A: not applicable.

#### Patient Datasets

Retrospective data will be available for all included patients (included population). Prospective data from the ePRO tool will be available for the subset of patients who complete the questionnaire (ePRO population).

#### Multilevel Models

Individuals living in the same country share a common health care system, lifestyle, and climate, and tend to be more similar than individuals from other countries. Therefore, individuals are considered nested within a country. This correlation of observations violates the assumption of independence for standard regression analysis leading to biased standard errors of parameter estimates. Therefore, we will fit multilevel linear or logistic mixed models using the country variable as a random effect to test the association between adherence or persistence and levels of service while accounting for this nested data structure.

#### Confounding Factors

Potential confounding factors were identified a priori based on a literature review and clinician expertise, and include age, sex, BMI, sleep duration, symptoms before starting PAP therapy, occupational status, highest education level, bed partner, comorbidities, smoking status, alcohol consumption, apnea-hypopnea index at the time of diagnosis, and prescriber specialty. Factors consistently described as being associated with PAP therapy adherence or persistence will be automatically included in statistical models comparing the effect of service on adherence or persistence. Other potential confounding factors will be selected based on their association with adherence or persistence in a univariate analysis. Full details of the confounding variables considered and the supporting sources are provided in Multimedia Appendix 2. Quantitative bias analysis will be performed to evaluate the impact of unmeasured confounders using a metric named the e-value [34]. The e-value can be defined as the strength of association that an unmeasured confounding factor would need to have in order to explain away the observed association.

#### Data Visualization

Data visualization (eg, with the use of Sankey diagrams) will be used to represent the pattern of changes in mean PAP use categories over time.

#### Sensitivity Analyses

Sensitivity analyses will be performed after removal of data collected during the COVID-19 lockdown periods, and before and after June 2021 (the time of the Philips Respironics world product recall [35,36].

### Ancillary Study

While the association between PAP adherence and costs has been demonstrated in the United States [21,23,37-42], data from Europe remain limited [43,44]. To further determine the benefits of home support providers, an ancillary study, named APAIR (Association Between Positive Airway Pressure Adherence, Morbi-Mortality and Costs Among Patients With Obstructive Sleep Apnea in France: Real World Study Using Claims and Telemonitoring Data), will be conducted in the subset of AWAIR study participants from France. APAIR will investigate the association between PAP adherence and persistence, and key outcomes (mortality, morbidity, and health-related costs). AWAIR study participants from France will be identified in the French Healthcare Claims Database (Système National des Données de Santé), which provides longitudinal patient-level data for approximately 99% of the French population [45]. Data on hospitalizations and sick leave related to the complications of OSA will be retrieved for these individuals. The aim of APAIR is to obtain data relating to new and more relevant thresholds for PAP adherence and the personalization of PAP therapy support (eg, for those with higher health risks or higher health care resource use).

## Results

As of December 2024, the AWAIR study has received approval in France, Portugal, and 2 regions of Spain. Patient enrollment started in France in October 2024, with around 90,000 patients informed about the study. Results of the AWAIR study are expected in the second quarter of 2025.

## Discussion

### Study Rationale

The high and increasing number of patients with OSA treated with PAP therapy drives concerns among health systems regarding the associated health care resource consumption and costs. This has led some countries to implement significant changes in the patient pathway, notably toward more home-centered models [30,46]. Regardless of the care setting, PAP adherence and persistence are essential because they are associated with health benefits [19], whereas poor adherence and PAP therapy terminations have been associated with increased disability, health care resource consumption, and direct or indirect costs [47-49].

Despite a large body of evidence regarding the factors associated with PAP adherence [50] and persistence [25,51], and the reimbursement of PAP-related services in many European Union countries, there is currently a lack of evidence regarding the impact of home support provider services on these outcomes. In addition, although defined by country or local regulations, the level of home support provider service is poorly described. Differences exist in service frequency, content, and communication channels between home support providers and patients, the use of PAP telemonitoring, and the qualifications of home support provider personnel. Some of these components have been shown to impact PAP adherence [29,30,52,53].

Our working hypothesis is that a higher level of home support provider service would be associated with better PAP adherence and persistence. This study will also investigate between-country differences in terms of patient characteristics, OSA-related characteristics, and services, and identify which components of service are most effective at improving adherence and persistence.

We anticipate that this research will provide valuable evidence to inform decision-making by authorities and payers regarding the regular reassessment of services and associated tariffs. It could also support guidelines relating to the follow-up of individuals receiving PAP therapy.

### Design Rationale

The comparative, real-world design with between-country comparisons was the most feasible approach for this study. A randomized controlled trial was not viable because home support provider services are implemented for all patients prescribed PAP. The need to include a control group without services in a randomized trial would have markedly reduced ethical approval and acceptance of the study and would have introduced important bias related to behavioral factors for those accepting home support provider management. Furthermore, the controlled conditions of clinical trials do not adequately capture the complexity of factors influencing adherence in real life. Furthermore, there was a need for evaluation of the real-world impact of home support providers on a representative, unselected population. Real-world studies provide information about outcomes with less rigid treatment patterns compared with a controlled trial [54] and bridge the gap between clinical research and everyday practice. We followed the Haute Autorité de Santé methodological recommendation for real-world studies, proposing a design consistent with identified questions, using preexisting high-quality data, integrating PROs and PRE, and ensuring transparency [55].

### Strengths

One of the major strengths of this study is its large sample size, which enhances the representativeness of the population and the generalizability of the findings. The retrospective design allows for long-term evaluation of PAP adherence and persistence and ensures that there is no influence on participants’ behavior because data were collected prior to their inclusion in the study. In addition, the AWAIR study will describe between-country differences in home provider services and patient characteristics for the first time, and report on patient satisfaction and experience with PAP therapy.

In contrast with conventional clinical trials, the real-world setting ensures that the results will reflect the usual course of care [56]. This is particularly important for evaluating adherence because it minimizes the risk of bias that could arise from study participation. Furthermore, real-world evidence is particularly relevant for meeting health authority post-approval effectiveness requirements [57]. Well-conducted comparative effectiveness research can provide valid results similar to randomized trials for this measure of known confounding factors [56], and can use methods (such as stratification, matching, or regression analyses) to account for or adjust for such factors and yield valid results [58]. In the real-world comparative AWAIR study, adjustment for potential confounders identified through literature review and clinical expertise, combined with the use of hierarchical models accounting for contextual differences related to the international nature of the study, will help the data to provide an accurate assessment of the net effect of home provider services on PAP adherence and persistence while minimizing the potential biases inherent in real-world studies. Additionally, the use of an ePRO tool facilitates the collection of data on confounding factors that would not otherwise be available in the retrospective database analysis, along with PROs, PREs, and health literacy across a large and diverse patient population from various geographic regions [59].

### Limitations

Selection bias cannot be entirely ruled out because participants were required to have a valid email address or smartphone number to be eligible for the study. This may lead to underrepresentation of certain subgroups of PAP users, such as older adults. Patients who answer the questionnaire might also differ from those who do not answer it. In addition, this study will only include individuals who use PAP supplied by a home support provider affiliated with ALH and it is possible that the level or content of additional services may differ slightly from other home support providers in the same country. To assess the potential impact of such biases, we will compare the characteristics of our study population with published data on OSA, focusing on age, sex, and disease severity.

Data from the ePRO tool may be subject to recall bias and inaccuracy because participants will complete the web-based questionnaire at variable periods of time since the start of PAP therapy (up to 5 years later for those who started PAP in 2019) and medical data will directly be provided by the patient. Confounding bias other than the key confounding factors considered is another limitation, particularly with respect to medical follow-up (data that will not be collected in this study).

Finally, the period over which this study was conducted included times before and during the COVID-19 pandemic (including lockdowns), and the worldwide recall of a PAP device made by Philips Respironics [36]. Sensitivity analyses will be performed to determine whether these factors had any influence on the overall study findings.

### Challenges

The study faced several challenges, including the regulatory classification of an ambispective study and the heterogeneous regulatory processes between countries. In France, the reuse of data for clinical research is facilitated by a simplified procedure that does not exist in other countries. Although the decentralized approach used in this study (ie, e-consent + ePRO tool) has advantages, especially in terms of managing a large number of participants, proposing the study to potential participants remotely can decrease the rate of enrollment. This is why a video-based approach was chosen because using videos to explain the research aims and procedures to potential participants of a clinical trial was preferred by patients over paper-based systems and improved their understanding [60].

Another challenge is the quantity of data to be processed due to the large number of participants and the daily PAP data collected over up to 5 years, requiring robust data management and quality control systems. Moreover, data were collected for routine home support provider services, in different countries with different services and enterprise resource planning systems, with different data collection processes, which adds another layer of complexity to the study. Therefore, those involved in the study need to have a good understanding of the data collection processes, data structures, formats, contents, and constraints of each system to allow standardization of data to constitute a research-ready database.

### Opportunities

Clinical trials are critical in the assessment of health products and services. However, managing and executing randomized controlled trials pose significant challenges in terms of inclusion, data collection, follow-up, comparator group, and costs [61]. Specifically in patients with OSA treated with PAP, it was shown that recruitment strategies in randomized trials could result in a lack of representativeness of the participants, because only up to 20% of typical patients with OSA meet the eligibility criteria, limiting the generalizability of the results [62]. The expanding availability of real-world data combined with advancements in operational tools and methodologies presents a significant opportunity to develop alternative study designs that leverage existing data. Furthermore, the European Health Data Space will create a common framework in Europe for the reuse of health data for research [63]. The design of the AWAIR study integrates multiple real-world data sources (including operational databases, device data, and an ePRO tool), the use of comparative real-world methods, and the implementation of e-consent. This innovative approach not only makes the study more robust but also sets a precedent for future research in similar fields, particularly regarding the number of participants who will consent and complete the ePRO tool. The design allows the study to be continuously enriched with data from new countries to measure the effect of other service levels and also to extend patient follow-up to assess longer-term persistence or include other patients in the countries of interest to assess the impact of changes in service levels.

### Conclusions

The AWAIR study has a unique design, leveraging an unprecedented number of eligible participants, decentralized technologies, and a real-world comparative methodology across multiple countries. This approach will highlight intercountry differences in terms of patient characteristics, PAP adherence and persistence, PROs, PREs, and satisfaction with home support provider services. By assessing the added value of home support provider services, the results will support best practices for patient management and decision-making by health authorities and payers.

## Appendix

Multimedia Appendix 1 Full study questionnaire.

Multimedia Appendix 2 Confounding factors.

## Abbreviations

ALH: Air Liquide Healthcare
APAIR: Association Between Positive Airway Pressure Adherence, Morbi-Mortality and Costs Among Patients With Obstructive Sleep Apnea in France: Real World Study Using Claims and Telemonitoring Data
AWAIR: Assessment of the Benefit of the Home Support Provider Service Level Associated With Positive Airway Pressure Treatment in Patients With Obstructive Sleep Apnea on Treatment Adherence and Persistence: an International Real-World Study
ePRO: electronic patient-reported outcome
ERP: Enterprise Resource Planning
HSP: homecare support provider
OSA: obstructive sleep apnea
PAP: positive airway pressure
PRE: patient-reported experience
PRO: patient-reported outcome
