# Supplementary material for: Evaluating the Benefit of Home Support Provider Services for Positive Airway Pressure Therapy in Patients With Obstructive Sleep Apnea: Protocol for an Ambispective International Real-World Study
Source: JMIR Res Protoc. 2025 Jan 31;14:e65840. doi: 10.2196/65840 (PMC11829180; doi:10.2196/65840)
Supplement: Multimedia Appendix 1 [file resprot_v14i1e65840_app1.docx]

**MULTIMEDIA APPENDIX FILE**

This is a Multimedia Appendix to a full manuscript published in the J Med Internet Res. For full copyright and citation information see http://dx.doi.org/10.2196/jmir.65840

**Appendix 1. Full study questionnaire**


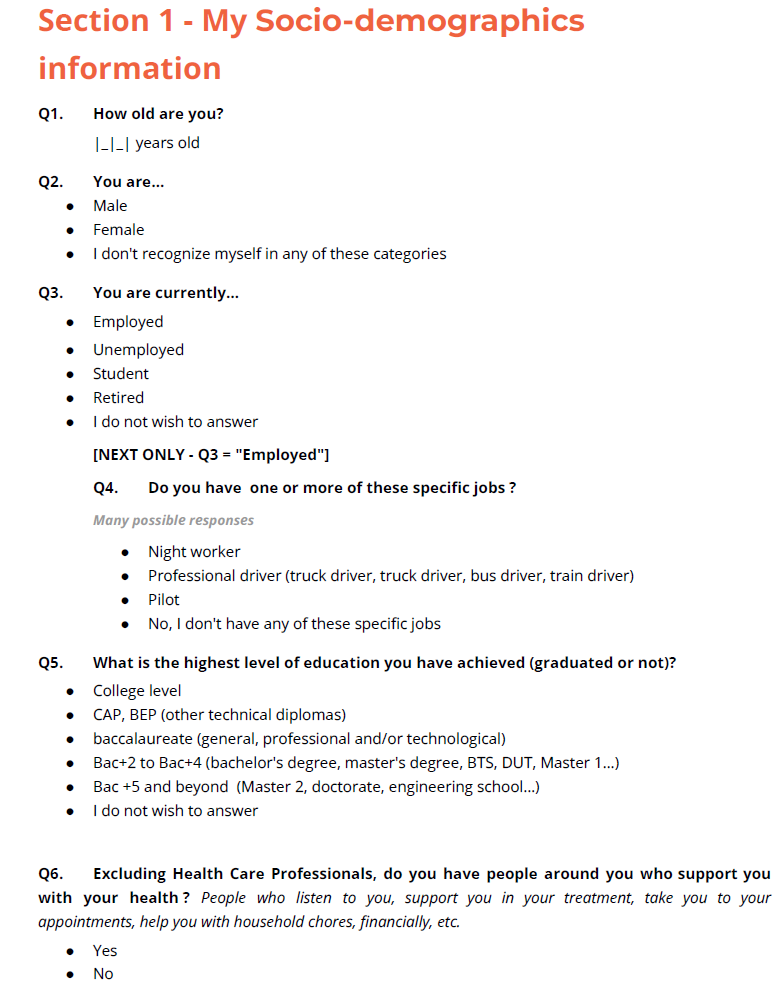


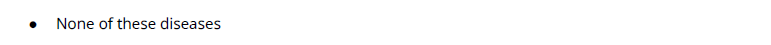

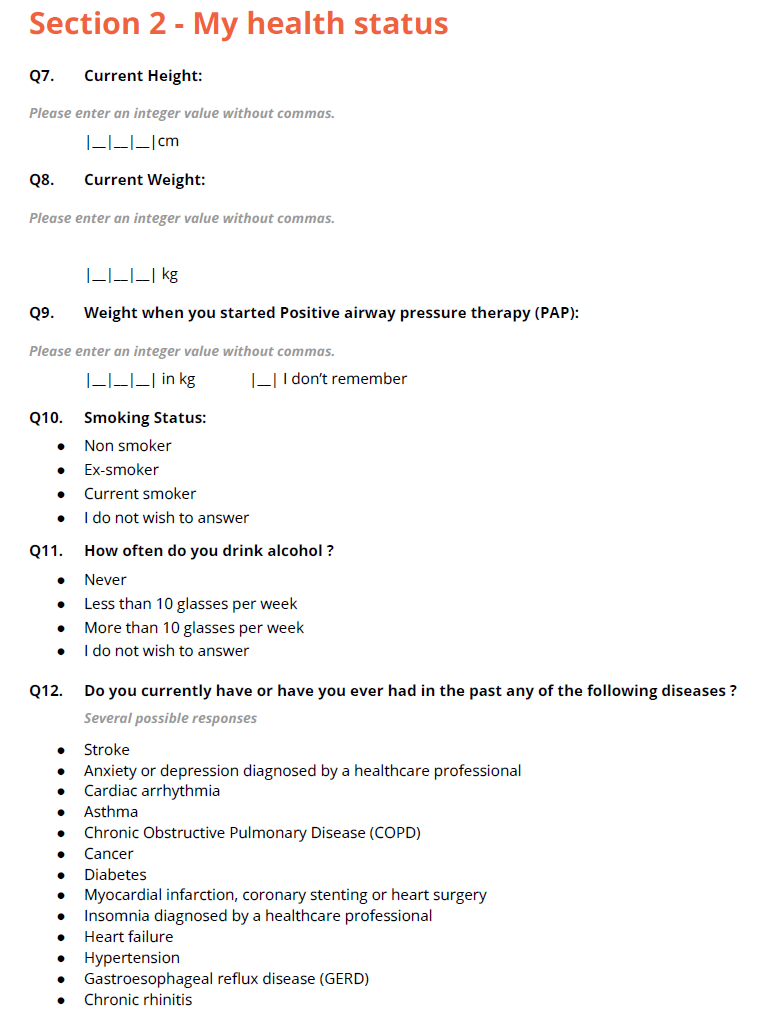


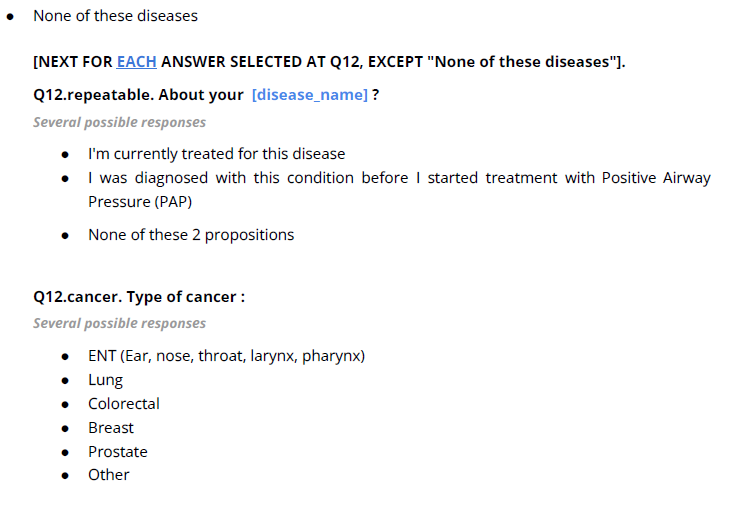


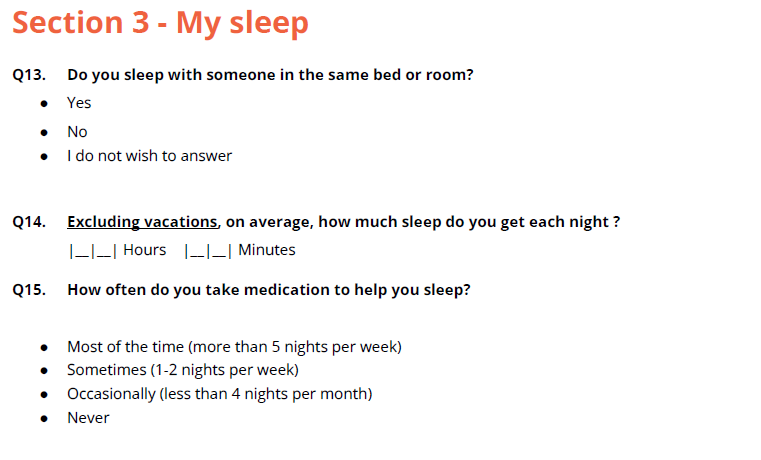


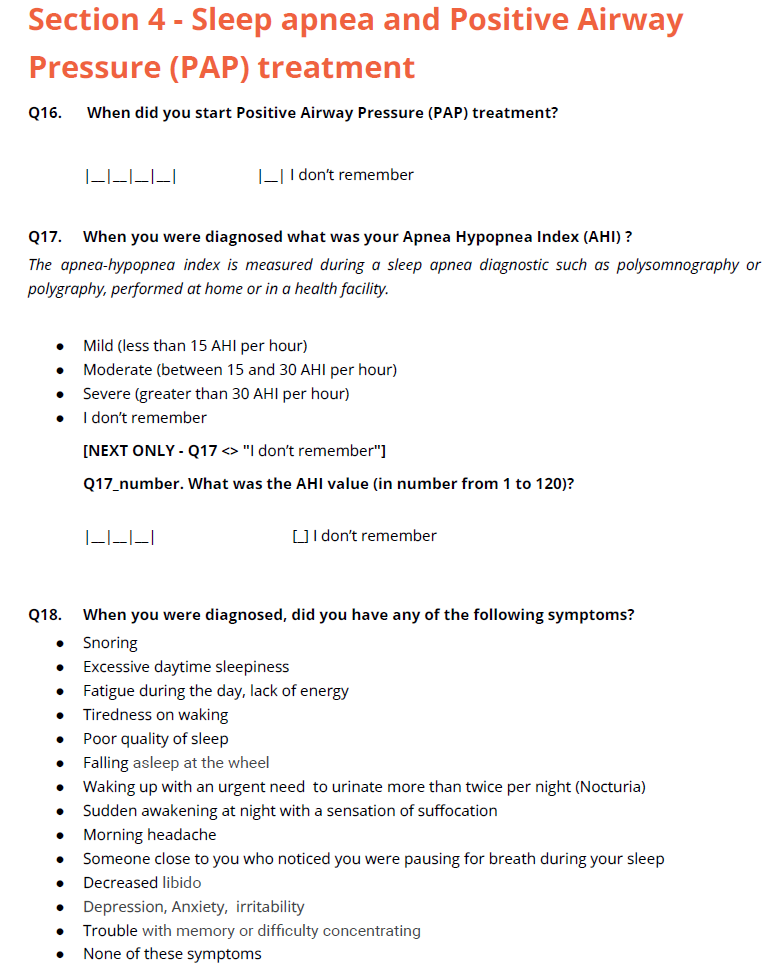


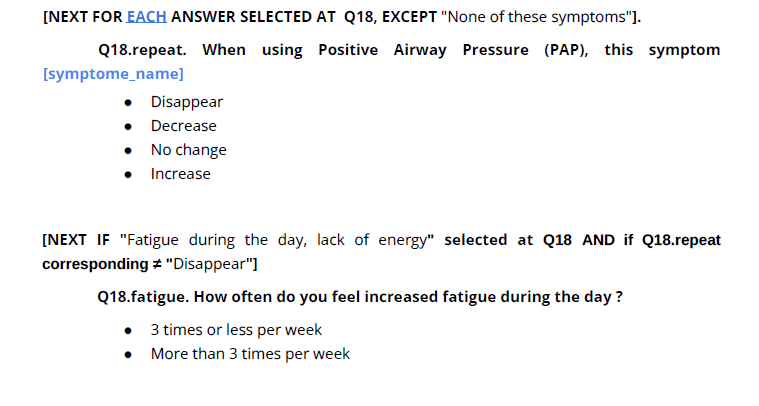


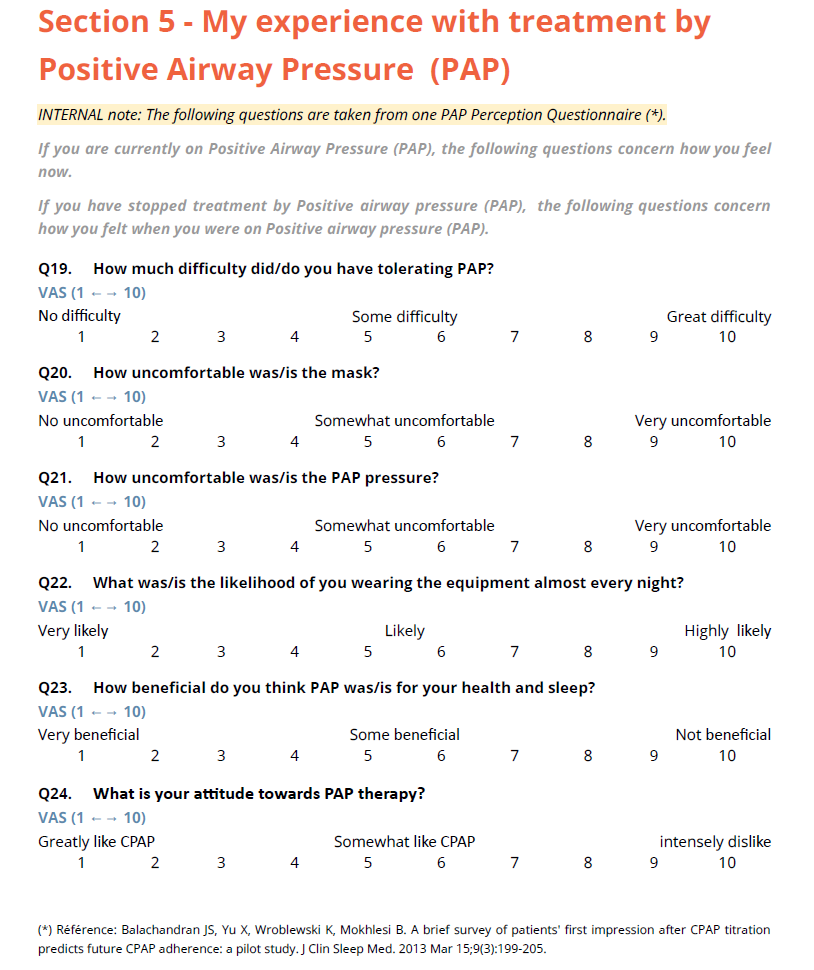


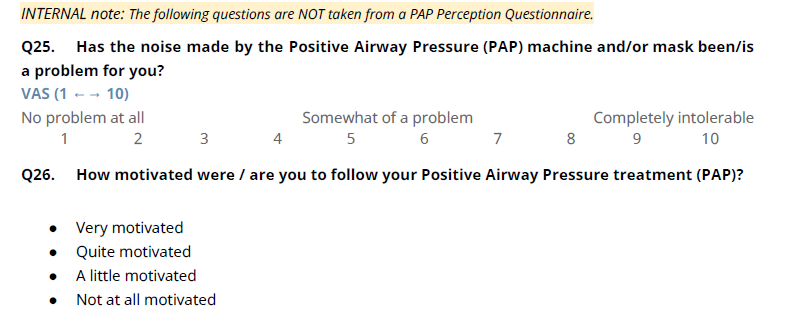


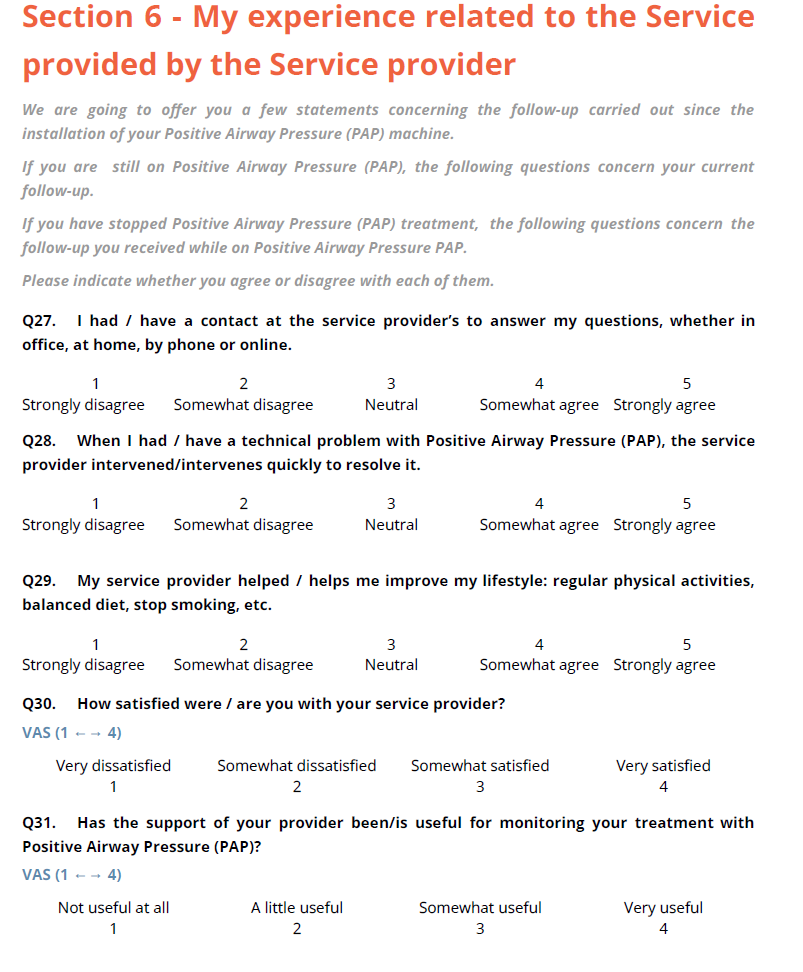


**
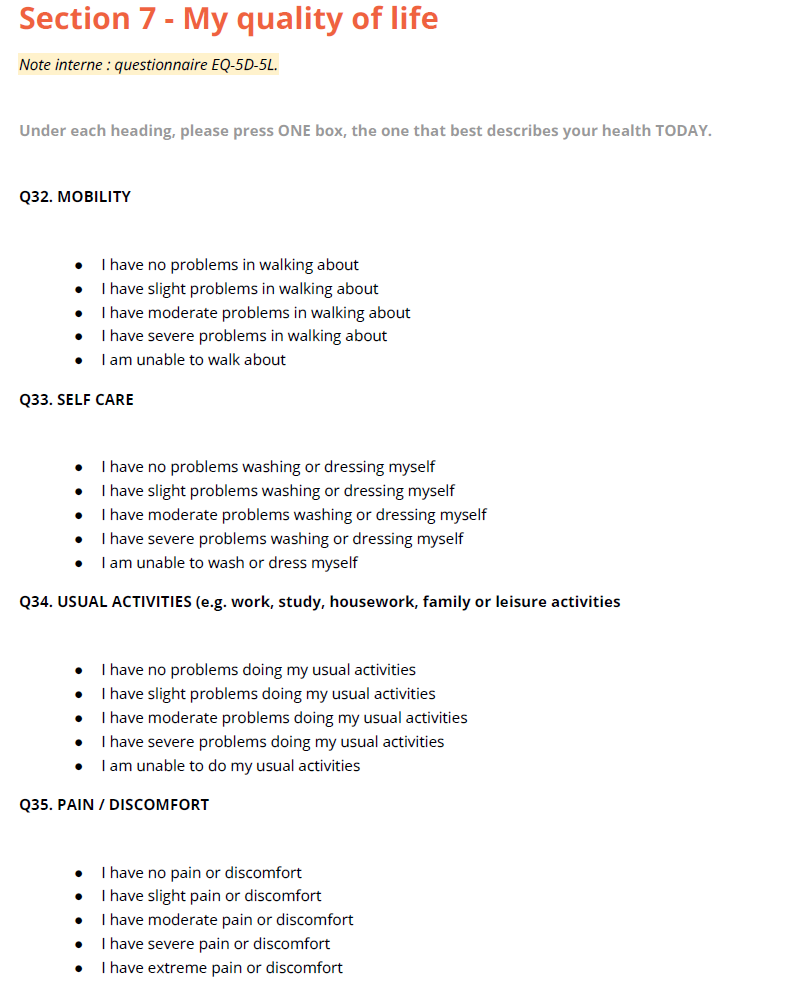
**

**
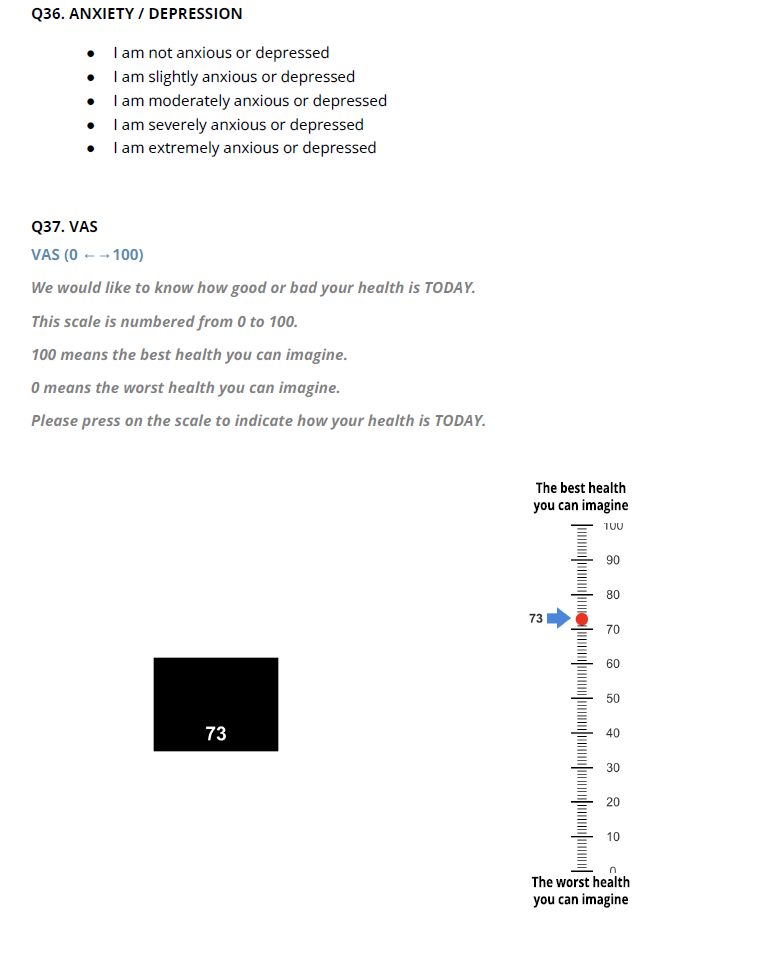
**

**
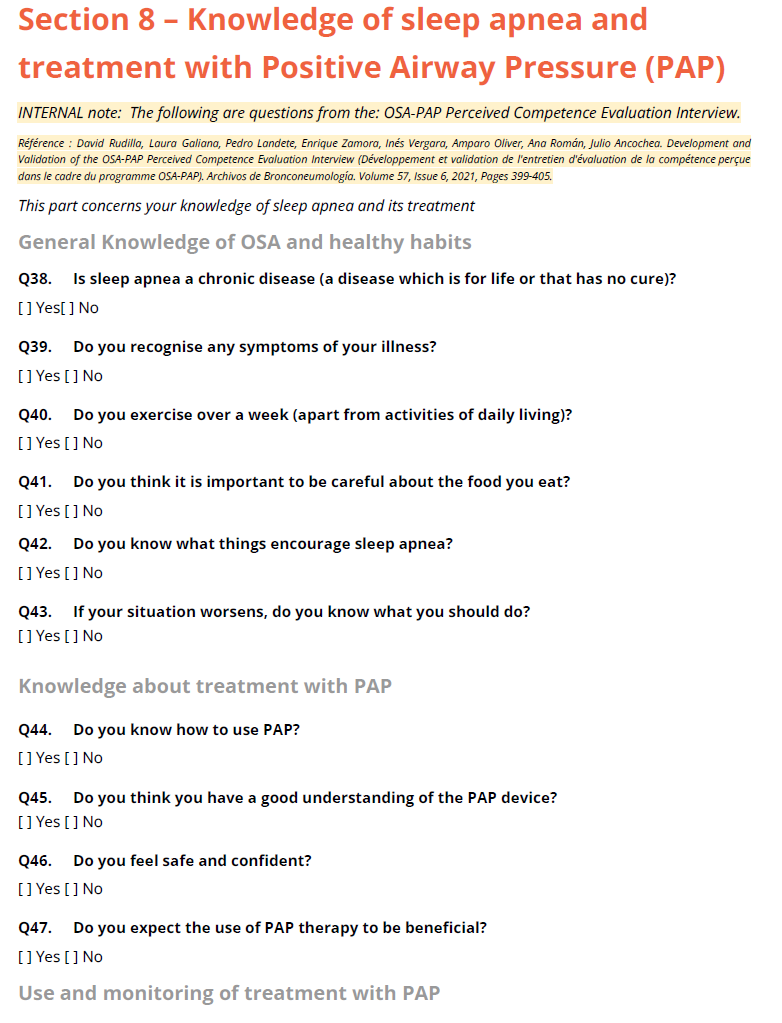
**

**
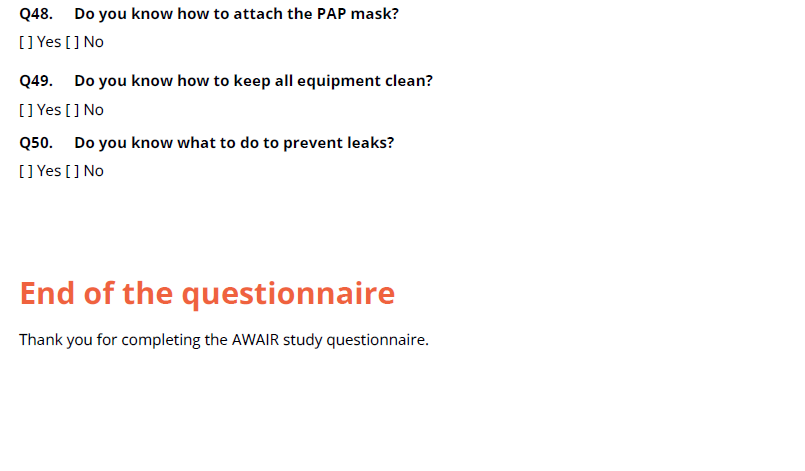
**
