# Supplementary material for: Evaluating the Benefit of Home Support Provider Services for Positive Airway Pressure Therapy in Patients With Obstructive Sleep Apnea: Protocol for an Ambispective International Real-World Study
Source: JMIR Res Protoc. 2025 Jan 31;14:e65840. doi: 10.2196/65840 (PMC11829180; doi:10.2196/65840)
Supplement: Multimedia Appendix 2 [file resprot_v14i1e65840_app2.docx]

**MULTIMEDIA APPENDIX FILE**

This is a Multimedia Appendix to a full manuscript published in the J Med Internet Res. For full copyright and citation information see http://dx.doi.org/10.2196/jmir.65840

**Appendix 2. Confounding factors**

Adherence and persistence to positive airway pressure (PAP) treatment can be influenced by several confounding factors. To assess the effect of the level of service on adherence/persistence with PAP therapy, these factors need to be identified and considered in the statistical analyses.

The following methodology was used to select the confounding factors to be considered in the statistical analyses:

- A pragmatic literature review was performed to identify relevant studies that highlighted associations between sociodemographic factors, health status, obstructive sleep apnea (OSA) severity and device-related factors and PAP adherence/persistence.
- A list of confounding factors was generated from the selected; factors measured after the start of the PAP therapy and that could be influenced either by the therapy or the service level were not retained.
- The results are presented in a summary table below where, for each factor, there is an explanation of the rationale its inclusion and the associated reference(s).
- Factors that are frequently described as being associated with adherence or persistence will be forced into the statistical models comparing the effect of service level on adherence or persistence.
- Other factors will be selected based on their association with adherence/persistence in a univariate analysis.

**Table S1.** Potential confounding factors relating to PAP adherence and persistence

| **Confounding factors** | **Conclusion from literature review** | **References** | **Forced in the model** |
| --- | --- | --- | --- |
| **Sociodemographic factors** |  |  |  |
| Age | Older age repeatedly associated with higher levels of PAP usage. There might be a U-shaped relationship between therapy termination and age; therapy termination was higher in the youngest and oldest patients (note: association between oldest age and therapy termination might be due to death) | Budhiraja et al 2007 [1]; Mehrtash et al 2019 [2]; Sin et al, 2019 [3]; Patel et al 2021 [4]; May et al 2023 [5]; Park et al 2023 [6]; Pépin et al 2021 [7]; Bonsignore et al 2023 [8]; Salpeci et al 2013 [9]; Woehrle et al 2018 [10] | Yes |
| Sex | Associations between sex and adherence found in the literature but results are inconsistent | Sin et al, 2019 [3]; Patel et al 2021 [4]; Pépin et al 2021 [7]; Bonsignore et al 2023 [8]; Woehrle et al 2018 [10]; Amfilochiou et al 2009 [11]; Joo et al 2007 [12] | Yes |
| Education | Socioeconomic status may play a role in adherence but the evidence is scarce and further research is needed regarding its effect. Higher income, educational level, and socioeconomic status have been associated with higher PAP adherence in some, but not all, studies | May et al 2023 [5]; Bonsignore et al 2023 [8]; Gulati et al 2017 [13]; Mendelson M et al 2023 [14] | No |
| Employment status |  |  | No |
| Night worker |  |  | No |
| Self-efficacy and risk perception | Low self-efficacy and lack of awareness regarding the risks of untreated OSA may play a role in adherence but the evidence is scarce and further research is needed regarding its effect | Archbold et al 2009 [15] | No |
| Bed partner | Effect in both directions: while the presence of a partner and support at home has an important positive influence on PAP compliance, it is also possible that PAP may bother a patient’s partner, leading to reduced intimacy, interference with their sexual relationship and non-adherence.  Note: data on this variable is collected in the questionnaire; we hypothesized that the status at the time of data collection was the same as at treatment initiation. | Bonsignore et al 2023 [8]; Lewis et al 2004 [16]; Sunwoo et al 2020 [17] | No |

| **Health status** |  |  |  |
| --- | --- | --- | --- |
| Comorbidities (diabetes, stroke, myocardial infarction, coronary stenting or heart surgery, heart failure, cardiac arrhythmia, hypertension, gastroesophageal reflux disease, COPD, asthma, allergic rhinitis, cancer, anxiety or depression, insomnia) | A large study (ALASKA, n=480 000) found that patients with ≥1 comorbidity have a higher risk of therapy termination, especially diabetes and COPD. Patients with hypertension were more likely to continue using PAP. Patients with diabetes were significantly more likely to terminate PAP after a first therapy resumption. Patients with comorbid insomnia and sleep apnea (COMISA) often have poor adherence to PAP therapy. | Pépin et al 2021 [7]; Bonsignore et al 2023 [8]; Lewis et al 2004 [16]; Sunwoo et al 2020 [17]; van Veldhuisen et al 2023 [18]; Stepanowsky et al 2002 [19]; Pieh et al 2013 [20]; Lack et al 2016 [21] | No |
| BMI | Inconsistent results: some studies found higher BMI to be a predictor of long‐term PAP use while obesity was associated with poor compliance in other studies. | Bonsignore et al 2023 [8]; van Veldhuisen et al 2023 [18]; Pépin et al 2024 [22]; Crew et al 2019 [23]; Gracia Hernández et al 2023 [24]; Jacobsen et al 2017 [25]; | Yes |
| Average sleep duration | Data on this variable is collected in the questionnaire; we hypothesized that sleep duration at the time of data collection was the same as at treatment initiation |  | Yes |
| Alcohol consumption | There is no evidence of an association between alcohol consumption and adherence, but this can be a marker of the healthy adherer effect.  Note: data on this variable was collected in the questionnaire; we hypothesized that alcohol consumption at the time of data collection was the same as at treatment initiation |  | No |
| Tobacco use | There is little evidence for an association between tobacco and adherence, but this can be a marker of the healthy adherer effect  Note: data on this variable was collected in the questionnaire; we hypothesized that tobacco use at the time of data collection was the same as at treatment initiation. | Jacobsen et al 2017 [25] | No |
| **OSA severity** |  |  |  |
| Symptoms at PAP initiation (snoring, excessive daytime sleepiness, fatigue during the day, lack of energy, waking up feeling tired, poor sleep, falling asleep at the wheel, nocturia, abrupt awakening with choking and gasping, morning headache, witnessed apneas during sleep, decreased libido, depression, anxiety, irritability, trouble with memory or difficulty concentrating) | Initial severity of daytime sleepiness has been associated with adherence | Bonsignore et al 2023 [8]; Sunwoo et al 2020 [17]; Jacobsen et al 2017 [25] | Yes (for EDS) |
| Use of hypnotic drugs | Patients who used hypnotic drugs were less likely to adhere to PAP therapy; this suggests that the use of sleep medications may interfere with the effectiveness or perceived need for PAP treatment | Jacobsen et al 2017 [25] | No |
| Initial AHI | OSA severity at diagnosis (based on the AHI) has not been recognized as a reliable predictor of PAP adherence | Bonsignore et al 2023 [8]; Sunwoo et al 2020 [17]; Sawyer et al 2011 [26] | No |
| **Device-related factors** |  |  |  |
| Type of mask | Lower adherence with oronasal versus nasal masks | Andrade et al 2014 [27] | No |
| Mask comfort | Discomfort with PAP masks can influence willingness to use the device | Sheth et al 2024 [28] | No |
| PAP device | Use of an automatically titrating PAP device was found to significantly improve compliance; this indicates that the type of equipment provided can influence a patient's willingness to use PAP regularly | Singhal et al 2016 [29] | No |
| **Pathway** |  |  |  |
| Prescriber | In the ALASKA study population who resumed PAP in the year after a first termination (n=27,212), device prescription by a pulmonologist versus general practitioner was a significant predictor of PAP continuation after resumption | Pépin et al 2024 [22] | No |

AHI, apnea-hypopnea index; BMI, body mass index; COPD, chronic obstructive pulmonary disease; EDS, excessive daytime sleepiness; OSA, obstructive sleep apnea; PAP, positive airway pressure therapy.

**References**

1. Budhiraja R, Parthasarathy S, Drake CL, Roth T, Sharief I, Budhiraja P et al. Early CPAP use identifies subsequent adherence to CPAP therapy. Sleep. 2007;30:320-324.

2. Mehrtash M, Bakker JP, Ayas N. Predictors of Continuous Positive Airway Pressure Adherence in Patients with Obstructive Sleep Apnea. Lung. 2019;197:115-121. doi:10.1007/s00408-018-00193-1.

3. Sin DD, Mayers I, Man GC, Pawluk L. Long-term compliance rates to continuous positive airway pressure in obstructive sleep apnea: a population-based study. Chest. 2002;121:430-435. doi:10.1378/chest.121.2.430.

4. Patel SR, Bakker JP, Stitt CJ, Aloia MS, Nouraie SM. Age and Sex Disparities in Adherence to CPAP. Chest. 2021;159:382-389. doi:10.1016/j.chest.2020.07.017.

5. May AM, Patel SR, Yamauchi M, Verma TK, Weaver TE, Chai-Coetzer CL et al. Moving toward equitable care for sleep apnea in the United States: positive airway pressure adherence thresholds: an official American Thoracic Society policy statement. Am J Respir Crit Care Med. 2023;207:244-254. doi:10.1164/rccm.202210-1846ST.

6. Park SI, Kim BK, Lee KE, Hong SD, Jung YG, Kim HY. Predictors for short-term and long-term automatic PAP compliance. J Clin Sleep Med. 2023;19:17-26. doi:10.5664/jcsm.10236.

7. Pépin JL, Bailly S, Rinder P, Adler D, Szeftel D, Malhotra A et al. CPAP therapy termination rates by OSA phenotype: a French nationwide database analysis. J Clin Med. 2021;10. doi:10.3390/jcm10050936.

8. Bonsignore MR, Randerath W, Schiza SE, Simonds AK. ERS Handbook of Respiratory Sleep Medicine. European Respiratory Society; 2023.

9. Salepci B, Caglayan B, Kiral N, Parmaksiz ET, Comert SS, Sarac G et al. CPAP adherence of patients with obstructive sleep apnea. Respir Care. 2013;58:1467-1473. doi:10.4187/respcare.02139.

10. Woehrle H, Arzt M, Graml A, Fietze I, Young P, Teschler H et al. Predictors of positive airway pressure therapy termination in the first year: analysis of big data from a German homecare provider. BMC Pulm Med. 2018;18:186. doi:10.1186/s12890-018-0748-8.

11. Amfilochiou A, Tsara V, Kolilekas L, Gizopoulou E, Maniou C, Bouros D et al. Determinants of continuous positive airway pressure compliance in a group of Greek patients with obstructive sleep apnea. Eur J Intern Med. 2009;20:645-650. doi:10.1016/j.ejim.2009.07.004.

12. Joo MJ, Herdegen JJ. Sleep apnea in an urban public hospital: assessment of severity and treatment adherence. J Clin Sleep Med. 2007;3:285-288.

13. Gulati A, Ali M, Davies M, Quinnell T, Smith I. A prospective observational study to evaluate the effect of social and personality factors on continuous positive airway pressure (CPAP) compliance in obstructive sleep apnoea syndrome. BMC Pulm Med. 2017;17:56. doi:10.1186/s12890-017-0393-7.

14. Mendelson M, Duval J, Bettega F, Tamisier R, Baillieul S, Bailly S et al. The individual and societal prices of non-adherence to continuous positive airway pressure, contributors, and strategies for improvement. Expert Rev Respir Med. 2023;17:305-317. doi:10.1080/17476348.2023.2202853.

15. Archbold KH, Parthasarathy S. Adherence to positive airway pressure therapy in adults and children. Curr Opin Pulm Med. 2009;15:585-590. doi:10.1097/MCP.0b013e3283319b3f.

16. Lewis KE, Seale L, Bartle IE, Watkins AJ, Ebden P. Early predictors of CPAP use for the treatment of obstructive sleep apnea. Sleep. 2004;27:134-138. doi:10.1093/sleep/27.1.134.

17. Sunwoo BY, Light M, Malhotra A. Strategies to augment adherence in the management of sleep-disordered breathing. Respirology. 2020;25:363-371. doi:10.1111/resp.13589.

18. van Veldhuisen SL, van Boxel MF, Wiezer MJ, van Veen RN, de Castro SMM, Swank DJ et al. Evaluation of CPAP adherence in bariatric patients diagnosed with obstructive sleep apnea: outcomes of a multicenter cohort study. Sleep Breath. 2023;27:535-544. doi:10.1007/s11325-022-02643-w.

19. Stepnowsky CJ, Jr., Bardwell WA, Moore PJ, Ancoli-Israel S, Dimsdale JE. Psychologic correlates of compliance with continuous positive airway pressure. Sleep. 2002;25:758-762. doi:10.1093/sleep/25.7.758.

20. Pieh C, Bach M, Popp R, Jara C, Crönlein T, Hajak G et al. Insomnia symptoms influence CPAP compliance. Sleep Breath. 2013;17:99-104. doi:10.1007/s11325-012-0655-9.

21. Lack L, Sweetman A. Diagnosis and Treatment of Insomnia Comorbid with Obstructive Sleep Apnea. Sleep Med Clin. 2016;11:379-388. doi:10.1016/j.jsmc.2016.05.006.

22. Pépin JL, Tamisier R, Benjafield AV, Rinder P, Lavergne F, Josseran A et al. CPAP resumption after a first termination and impact on all-cause mortality in France. Eur Respir J. 2024;63. doi:10.1183/13993003.01171-2023.

23. Crew EC, Wohlgemuth WK, Wallace DM. Improving adherence to PAP therapy: an update. Curr Pulmonol Rep. 2019;8:1-13. doi:10.1007/s13665-019-0220-0.

24. Gracia Hernández B, González Hernández A, Rodríguez Gómez AB, Hilares Vera JI. Factors influencing the CPAP therapeutic adherence in women with obstructive sleep apnea (OSA). Eur Respir J. 2023;62:PA577. doi:10.1183/13993003.congress-2023.PA577.

25. Jacobsen AR, Eriksen F, Hansen RW, Erlandsen M, Thorup L, Damgård MB et al. Determinants for adherence to continuous positive airway pressure therapy in obstructive sleep apnea. PLoS One. 2017;12:e0189614. doi:10.1371/journal.pone.0189614.

26. Sawyer AM, Gooneratne NS, Marcus CL, Ofer D, Richards KC, Weaver TE. A systematic review of CPAP adherence across age groups: clinical and empiric insights for developing CPAP adherence interventions. Sleep Med Rev. 2011;15:343-356. doi:10.1016/j.smrv.2011.01.003.

27. Andrade RG, Piccin VS, Nascimento JA, Viana FM, Genta PR, Lorenzi-Filho G. Impact of the type of mask on the effectiveness of and adherence to continuous positive airway pressure treatment for obstructive sleep apnea. J Bras Pneumol. 2014;40:658-668. doi:10.1590/s1806-37132014000600010.

28. Sheth R, Audette M, Sheth S. Positive airway pressure and mask factors affecting adherence in patients with obstructive sleep apnea. SVOA Neurol. 2024;5:132-135.

29. Singhal P, Joshi Y, Singh G, Kulkarni S. Study of factors affecting compliance of continuous positive airway pressure (CPAP) in obstructive sleep apnea-hypopnea syndrome (OSAHS). Eur Respir J. 2016;48:PA2362. doi:10.1183/13993003.congress-2016.PA2362.
